# INVESTIGATING THE EFFECTS OF PM2.5 ON DEMENTIA RISK: A CASE-CONTROL SWEDISH TWIN REGISTRY STUDY

**DOI:** 10.1093/geroni/igad104.3442

**Published:** 2023-12-21

**Authors:** Luisa Parkinson, Ida Karlsson, Malin Ericsson, Sam Tomlinson, Finn Lindgren, Tom Russ, Deborah Finkel, LifeGEIST consortium

**Affiliations:** University of Edinburgh, Edinburgh, Scotland, United Kingdom; Karolinksa Institutet, Stockhoolm, Stockholms Lan, Sweden; Karolinksa Institutet, Stockhoolm, Stockholms Lan, Sweden; UK Centre for Ecology and Hydrology, Lancaster, England, United Kingdom; University of Edinburgh, Edinburgh, Scotland, United Kingdom; University of Edinburgh, Edinburgh, Scotland, United Kingdom; University of Southern California, Los Angeles, California, United States; University of Southern California, Los Angeles, California, United States

## Abstract

There is growing evidence that air pollution, and particularly PM2.5, is associated with dementia risk around the time of diagnosis. However few studies are able to investigate longer term effects of air pollution due to a lack of historical air pollution data. In this study we use PM2.5 data for Sweden available at 5-yearly intervals to investigate the short and longer term effects of PM2.5 on dementia risk. A case-control data set from the Swedish Twin Registry with dementia ascertained from hospital admissions between 1990 and 2015 and municipality of residence available at time of admission was analysed using hierarchical spatial models with R-INLA. The analyses show a relative risk of dementia per 5µg/m3 PM2.5 of 1.43 (95% confidence interval: 1.13-1.80), 1.36 (1.14-1.64), 1.31 (1.11-1.54) and 1.04 (1.00-1.09) for 0-5, 6-10, 11-15 and 16-20 years prior to diagnosis respectively. The cumulative effect over 0-20 years prior to diagnosis of an annual 5µg/m3 increase in PM2.5 on the relative risk of dementia is 1.19 (1.06-1.34). The clear association between PM2.5 and dementia risk for up to 20 years prior to diagnosis suggests that there are long term effects of air pollution on dementia risk. For these analyses only one residential location was available, so the uncertainty about an individual’s residential location increases with time prior to diagnosis. To fully understand the effects of air pollution on dementia risk a life-course approach in a data set with lifetime residential history and access to historical air pollution data will be necessary.

